# Deletion of biosynthetic genes, specific SNP patterns and differences in transcript accumulation cause variation in hydroxynitrile glucoside content in barley cultivars

**DOI:** 10.1038/s41598-019-41884-w

**Published:** 2019-04-05

**Authors:** Marcus Ehlert, Lea Møller Jagd, Ilka Braumann, Christoph Dockter, Christoph Crocoll, Mohammed Saddik Motawia, Birger Lindberg Møller, Michael Foged Lyngkjær

**Affiliations:** 1 Plant Biochemistry Laboratory, Department of Plant and Environmental Sciences, University of Copenhagen, Thorvaldsensvej 40, 1871 Frederiksberg C, Copenhagen Denmark; 2 VILLUM Research Center for Plant Plasticity, University of Copenhagen, Thorvaldsensvej 40, 1871 Frederiksberg C, Copenhagen Denmark; 3 Carlsberg Research Laboratory, J.C. Jacobsens Gade 4, 1799 Copenhagen V, Denmark; 4 DynaMo Center, Department of Plant and Environmental Sciences, University of Copenhagen, Thorvaldsensvej 40, 1871 Frederiksberg C, Denmark

## Abstract

Barley (*Hordeum vulgare* L.) produces five leucine-derived hydroxynitrile glucosides, potentially involved in alleviating pathogen and environmental stresses. These compounds include the cyanogenic glucoside epiheterodendrin. The biosynthetic genes are clustered. Total hydroxynitrile glucoside contents were previously shown to vary from zero to more than 10,000 nmoles g^−1^ in different barley lines. To elucidate the cause of this variation, the biosynthetic genes from the high-level producer cv. Mentor, the medium-level producer cv. Pallas, and the zero-level producer cv. Emir were investigated. In cv. Emir, a major deletion in the genome spanning most of the hydroxynitrile glucoside biosynthetic gene cluster was identified and explains the complete absence of hydroxynitrile glucosides in this cultivar. The transcript levels of the biosynthetic genes were significantly higher in the high-level producer cv. Mentor compared to the medium-level producer cv. Pallas, indicating transcriptional regulation as a contributor to the variation in hydroxynitrile glucoside levels. A correlation between distinct single nucleotide polymorphism (SNP) patterns in the biosynthetic gene cluster and the hydroxynitrile glucoside levels in 227 barley lines was identified. It is remarkable that in spite of the demonstrated presence of a multitude of SNPs and differences in transcript levels, the ratio between the five hydroxynitrile glucosides is maintained across all the analysed barley lines. This implies the involvement of a stably assembled multienzyme complex.

## Introduction

Bioactive natural products (specialized metabolites) are present in all plant species. Although not essential for plant growth and development, they offer beneficial functions to the plant, for instance in terms of increased tolerance to biotic and abiotic stresses such as herbivore and pathogen attack, drought, or heat. α-, β- and γ-Hydroxynitrile glucosides are a widespread and ancient class of bioactive natural products and are found in pteridophytes, angiosperms, and gymnosperms^1^. The α-hydroxynitrile glucosides are also termed cyanogenic glucosides, due to their potential to release toxic hydrogen cyanide. Their involvement in plant chemical defence has been subject to numerous reviews^2–4^. To avoid autotoxicity, the cyanogenic glucoside is stored apart from its hydrolysing β-glucosidase^3–6^. Tissue disruption, e.g. caused by chewing insects, disintegrates the spatial separation and the β-glucosidase hydrolyses the cyanogenic glucoside, resulting in the release of hydrogen cyanide. In addition to their function as defence compounds, cyanogenic glucosides serve as mobilizable reservoirs of reduced carbon and nitrogen^7–10^. Two independent recycling pathways have been reported both involving turnover of the cyanogenic glucoside with a nitrile compound as an intermediate^7–9^. Nitrilase catalysed metabolism of the nitrile proceeds via an amide, which is hydrolysed into the corresponding carboxylic acid with concomitant release of ammonium^7^. In cassava, the recycling process shows a diurnal rhythm with maximum turn-over during the light period resulting in a 20% decrease in the total cyanogenic glucoside content^10^. This offers a source of reduced nitrogen to balance photosynthetic carbon fixation at high light irradiation. Rapid decreases in cyanogenic glucoside content following light spikes suggest an additional function as scavengers of reactive oxygen species^3,10^. Cyanogenic glucosides may co-exist with structurally related, non-cyanogenic β- and γ-hydroxynitrile glucosides, which may also have defence functions^2^.

In barley (*Hordeum vulgare* L.), five hydroxynitrile glucosides have been identified^11–13^: the cyanogenic α-hydroxynitrile glucoside epiheterodendrin, the β-hydroxynitrile glucoside epidermin, and the γ-hydroxynitrile glucosides dihydroosmaronin, osmaronin, and sutherlandin. Unlike most other cyanogenic plants, no specific β-glucosidase is present in barley leaves^13^, and consequently β-glucosidase-based detonation of a defence system based on a hydrogen cyanide bomb is not operating in barley leaves. The five hydroxynitrile glucosides are derived from the amino acid L-leucine. The biosynthesis of the barley hydroxynitrile glucosides has recently been elucidated and involves multifunctional cytochrome P450 (CYP) enzymes of the CYP79 and CYP71 families and UDP-glucosyltransferases (UGTs) of the UGT85 family^14^. The biosynthetic genes were found to be organized in a gene cluster^14^. Such clusters were previously also reported in lotus (*Lotus japonicus*), sorghum (*Sorghum bicolor*), and cassava (*Manihot esculenta*)^15^ but are absent in almond (*Prunus dulcis*)^16^ and sugar gum (*Eucalyptus cladocalyx*)^17^. The parent amino acid is initially converted into (*E*)-3-methylbutanal oxime by either CYP79A12 or CYP79A8. Subsequent hydroxylations and dehydrations of the oxime are carried out by a set of CYP71 enzymes (CYP71L1, CYP71C103, CYP71C113, and CYP71U5). The resulting hydroxynitriles are glucosylated by UGT85F22 or UGT85F23^14^. Barley hydroxynitrile glucosides are *de novo* synthesized in germinating seeds and young shoots^13^, where they accumulate in the leaf epidermis cells constituting for up to 90% of the soluble carbohydrates^12^. Analysis of the total contents in seedlings of 247 spring barley lines including landraces and old and modern cultivars revealed cultivar-specific hydroxynitrile glucoside contents between zero and more than 10,000 nmoles g^−1^ fresh weight, while the ratios between the compounds remained almost constant^14^.

The cyanogenic epiheterodendrin is an undesired constituent in barley malt used for the production of whisky. Epiheterodendrin was identified as the source of the carcinogenic contaminant ethyl carbamate^18,19^. Epiheterodendrin is hydrolysed by a yeast β-glucosidase during fermentation, resulting in the formation of isobutyraldehyde cyanohydrin. Upon its dissociation, hydrogen cyanide is formed and reacts with ethanol during the distillation process in copper vessels resulting in ethyl carbamate formation^18–21^. Today, the level of ethyl carbamate in whisky is efficiently controlled using cultivars producing zero or low amounts of epiheterodendrin^18,19,21^. However, in such cultivars, the linked biosynthesis of α-, β- and γ-hydroxynitrile glucosides may result in unintended selection against otherwise potentially beneficial β- and γ-hydroxynitrile glucosides with respect to herbivore and pathogen defence^3,4,22,23^.

In this study, we investigated whether the variation between the total hydroxynitrile glucoside contents in different barley cultivars can be explained by cultivar-specific polymorphisms in the biosynthetic genes, or by their differential transcriptional regulation. A deletion spanning most of the hydroxynitrile glucoside biosynthetic gene cluster was identified in cv. Emir and explains why this cultivar does not produce any hydroxynitrile glucosides. The hydroxynitrile glucoside levels in 227 barley lines were shown to correlate with distinct single nucleotide polymorphism (SNP) patterns in the biosynthetic gene cluster. Gene expression analysis combined with the quantification of the total hydroxynitrile glucoside contents in cv. Pallas and cv. Mentor shows that higher gene expression levels result in increased compound levels, indicating that transcriptional regulation is involved in the determination of the total hydroxynitrile glucoside content.

## Results

### Revised gene cluster

**Figure 1:**
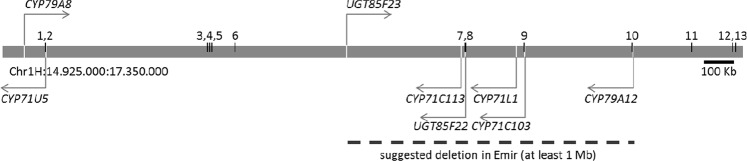
Genomic organization of the barley hydroxynitrile glucoside biosynthetic gene cluster on chromosome 1H. Arrows indicate orientation on the forward (right) or reverse (left) strand. Numbers above the cluster indicate SNP marker positions listed in Supplementary Fig. S1. Kb: kilobase. Mb: megabase. Accession numbers: *CYP79A8*, HORVU1Hr1G007400; *CYP71U5*, HORVU1Hr1G007420; *UGT85F23*, HORVU1Hr1G007720; *CYP71C113*, HORVU1Hr1G007790; *UGT85F22*, HORVU1Hr1G007810; *CYP71L1*, HORVU1Hr1G007830; *CYP71C103*, HORVU1Hr1G007840; *CYP79A12*, HORVU1Hr1G007900.

Blastn searches against the revised barley cv. Morex genome^24^ using the sequences of the genes encoding hydroxynitrile glucoside biosynthetic enzymes previously described by Knoch *et al*.^14^ identified 100% identical sequences located in close proximity to each other on barley chromosome 1 H. In the new genome assembly, the two CYP79 genes (*CYP79A8* and *CYP79A12*) are located at each end of the cluster, and the *CYP79A8* and *CYP71U5* genes are situated 1 Mb downstream of the other six genes, which are found within a 1 Mb sequence segment. Based on the revised barley genome sequence, Fig. 1 presents a revised configuration of the gene cluster reported in Knoch *et al*.^14^.

### Association between haplotypes and hydroxynitrile glucoside contents

Based on the hybridization patterns of 13 single nucleotide polymorphism (SNP) markers located within or in close proximity to the gene cluster, we identified 19 different haplotypes among the 227 different barley lines with hydroxynitrile glucoside contents known to vary from zero to more than 10,000 nmoles g^−1^ fresh mass^14^ (Supplementary Fig. S1). The SNP marker analysis revealed an association between some of the 19 haplotypes and their total hydroxynitrile glucoside production volume. Interestingly, 10 barley lines were missing hybridization signals to 4 markers (7, 8, 9 and 10) and 2 lines to 3 markers (7, 8 and 9 or 8, 9 and 10) of the SNP markers located in the central part of the gene cluster (Fig. 1). All 12 barley lines with missing hybridization signals are modern cultivars or breeding lines, and in all cases, the missing hybridization correlated with zero-production of hydroxynitrile glucosides.

### The biosynthetic genes are partially deleted in cv. Emir and show unique SNPs in cvs. Pallas and Mentor

Several SNPs were identified in the genomic DNA of the biosynthetic genes derived from the three cvs. Emir, Pallas, and Mentor when compared to the barley reference genome based on cv. Morex^24^. Interestingly, we were unable to PCR-amplify and hence sequence most of the biosynthetic genes in cv. Emir, indicating a deletion within the gene cluster spanning at least 1 Mb. This deletion included the genes encoding UGT85F23, CYP71C113, UGT85F22, CYP71L1, CYP71C103, and CYP79A12, and spans the same region of the gene cluster where the mentioned SNP marker hybridization signals in the zero-hydroxynitrile glucoside producers are missing (Fig. 1).

A total of 11, 16, and 29 SNPs positioned inside the open reading frames were found in the cvs. Emir, Pallas, and Mentor, respectively (summarized in Table 1). None of these were located within the predicted substrate recognition sites, cytochrome P450 cysteine heme-iron ligand signatures, or UDP-glycosyltransferase signatures. The first and potentially rate-determining step in the biosynthesis of hydroxynitrile glucosides is catalysed by one of the two CYP79 enzymes. For CYP79A12, the amino acid sequence of the first 61 positions in cvs. Pallas and Mentor additionally differed in 13 positions from the reference sequence of cv. Morex (Table 1 and Supplementary Fig. S2). Alignment with available genome sequences from cvs. Haruna Nijo, Bowman, and Bonus showed that the cultivars separated into two groups (cvs. Morex and Haruna Nijo and cvs. Pallas, Mentor, Bowman, and Bonus), each with distinct sequence changes within the first 61 amino acid positions. In the remaining part of the protein sequence, four additional amino acid substitutions were identified, with three being shared between cvs. Pallas and Mentor, and one substitution (P337Q in the protein, listed as position 442 in the alignment) that was exclusive for cv. Mentor. For CYP79A8, three shared substitutions between cvs. Emir and Mentor were identified. The CYP71 enzymes catalyse the conversion of the E-oxime intermediate into the final hydroxynitrile aglucons. Except for CYP71U5, only few substitutions were identified in the CYP71s. CYP71L1 was highly conserved and only a single amino acid substitution was identified in the cvs. Pallas and Mentor. In CYP71C103, a single substitution was identified in cv. Pallas, and four other substitutions in cv. Mentor. In CYP71C113, three substitutions were identified in cv. Pallas and two in cv. Mentor. More variation was observed in CYP71U5 where seven amino acid substitutions shared between cvs. Emir and Mentor were identified. One of these was also found in cv. Pallas. In addition, cv. Pallas carried another unique substitution. The UGT85 family enzymes catalyse the final step in the biosynthesis, the glycosylation of the hydroxynitrile aglucons. Several substitutions were identified in UGT85F22. Three substitutions were shared between cvs. Pallas and Mentor, and cv. Pallas carried two and cv. Mentor three additional unique substitutions. In UGT85F23, a single substitution shared between the cvs. Pallas and Mentor was identified.

**Table 1:** Polymorphisms in the hydroxynitrile glucoside biosynthesis genes and resulting amino acid substitutions.

Gene name	Cultivar	Mutation in cds	Amino acid substitution
*CYP79A12*	P, M	1–168	1–56 (several; more similarity to Bowman)
	P, M	220A → G	N74D
	P, M	510T → G	I170M
	P, M	712A → G	I238V
	M	1010C → A	P337Q
*CYP79A8*	E, M	473C → G	A158G
	E, M	996G → C	K332N
	E, M	1016G → A	R339Q
*CYP71L1*	P, M	127A → G	N43D
*CYP71U5*	E, M	419A → C	Q1450P
	E, P, M	833T → C	V278A
	P	896A → G	N299S
	E, M	1060G → A	V354I
	E, M	1264G → A	G422S
	E, M	1402C → A	H468N
	E, M	1426G → A	E476T
	E, M	1427A → C	E476T
	E, M	1441C → G	Q481E
*CYP71C103*	M	554G → C	S185T
	M	1059C → A	D353E
	P	1163T → C	I388T
	M	1235G → C	S412T
	M	1448A → C	E483A
*CYP71C113*	P	23A → G	E8G
	M	74T → C	M25T
	P	88G → A	G30R
	M	121C → T	R41C
	P	1168G → A	V390I
*UGT85F22*	P, M	315G → C	Q105H
	M	608C → T	T203I
	P, M	679A → G	I227V
	P, M	748C → A	P250T
	P	1334A → G	E445G
	M	1345G → A	G449R
	P	1397C → G	A466G
	M	1435G → A	V479I
*UGT85F23*	P, M	1489C → T	P497S

Numbers indicate positions in the coding sequence and protein, and the mutations are in relation to the sequences in cv. Morex. Only mutations located within exons and causing an amino acid substitution in the mature proteins are shown. E, Emir; P, Pallas; M, Mentor.

### Early hydroxynitrile glucoside biosynthesis and conserved compound ratios

**Figure 2:**
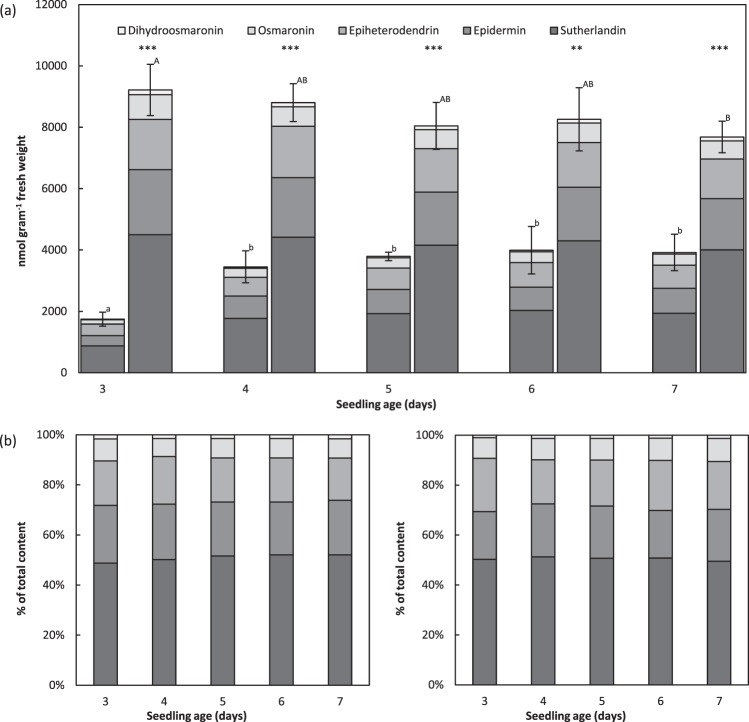
Hydroxynitrile glucoside contents in the cvs. Pallas and Mentor over time and ratios between the compounds. (**a**) Total contents over time in shoot tissue of the cvs. Pallas and Mentor (data shown as means ± s.d.). Asterisks indicate significantly different total contents between the cvs. Pallas and Mentor (***p < 0.001, **p < 0.01). Significantly different groups (p at least < 0.05) within the cultivars are indicated by small letters (Pallas) and capital letters (Mentor). (**b**) Ratio of the individual compounds in percentage of the total content from cv. Pallas (left) and cv. Mentor (right) at each time point.

Analysis of the total hydroxynitrile glucoside contents in shoot tissue from the cvs. Pallas and Mentor, sampled between 3 and 7 days after seed wetting, confirmed that cv. Pallas produces medium amounts (approximately 4,000 nmol g^−1^ fresh mass) and cv. Mentor produces high amounts (approximately 8,000 nmol g^−1^ fresh mass) of hydroxynitrile glucosides (Fig. 2a). In cv. Pallas, the content of hydroxynitrile glucosides increased strongly between day 3 and 4 and remained constant for the rest of the period. In contrast, cv. Mentor already accumulated high levels at day 3, which gradually decreased over time. Compared to cv. Pallas, the total content in cv. Mentor was significantly higher at all time points. The ratio between the five compounds always remained constant (Fig. 2b).

### Upregulated biosynthetic genes in the high-level producer cv. Mentor

**Figure 3:**
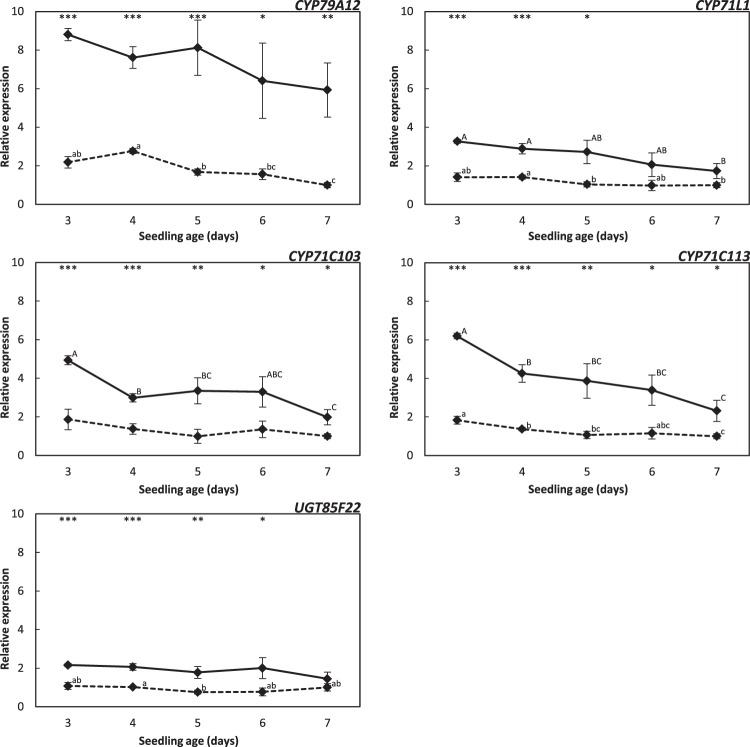
Relative expression of hydroxynitrile glucoside biosynthesis genes over time in shoot tissue of the cvs. Pallas (dashed lines) and Mentor (solid lines). The expression levels were normalized to three reference genes. Cultivar-specific relative expression levels were calculated by comparison to day seven as reference (data shown as means ± s.e.). Gene-specific expression levels and standard errors from cv. Mentor were corrected with a multiplication factor calculated by comparison of the expression levels between the cultivars at day seven. Asterisks indicate significantly different expression levels between the cvs. Pallas and Mentor (***p < 0.001, **p < 0.01, *p < 0.05). Significantly different groups (p at least <0.05) within the cultivars are indicated by small letters (Pallas) and capital letters (Mentor).

The majority of the genes encoding the biosynthetic enzymes for hydroxynitrile glucoside formation is deleted in cv. Emir and no hydroxynitrile glucosides are produced in this cultivar. Gene expression was therefore only examined in the cvs. Pallas and Mentor (Fig. 3). Except for the *CYP71L1* gene at day 6 and 7 and the *UGT85F22* gene at day 7, the five biosynthetic genes analysed were significantly higher expressed in cv. Mentor than in cv. Pallas at all time points, and cv. Mentor produced higher amounts of hydroxynitrile glucosides. The largest difference was observed for the *CYP79A12* gene, encoding the first committed enzyme of the hydroxynitrile glucoside biosynthetic pathway. In general, differences in the expression of the same genes in the different cultivars were higher at the early time points and decreased over the time period. Within the cultivars, expression of the biosynthetic genes was higher at the early time points and decreased over the period. This was most prominent in cv. Mentor. The lowest regulation was observed for the *UGT85F22* gene, which retained a constant expression level within both cultivars.

## Discussion

Re-sequencing of the genes for hydroxynitrile glucoside biosynthesis in the zero-level producer cv. Emir, the medium-level producer cv. Pallas, and the high-level producer cv. Mentor, and also the missing hybridization to SNP markers in the biosynthetic genes in other zero-level producers, demonstrated that a deletion of at least 1 Mb within the biosynthetic gene cluster is responsible for the lost ability to produce hydroxynitrile glucosides in cv. Emir (Fig. 1). The cv. Emir was previously reported to produce low amounts of hydroxynitrile glucosides^13,25^. However, no hydroxynitrile glucosides were identified in cv. Emir in this study, using a purified seed batch. Zero-production in cv. Emir was also reported by Swanston *et al*.^21^, who suggested that an *Eph* locus on chromosome 1 H is responsible for epiheterodendrin biosynthesis, and that the zero *eph* allele in cv. Emir originated from cv. Arabische^21^. The zero *eph* allele was further found in the other tested cultivars lacking epiheterodendrin, which all had cv. Emir in their pedigree^21^. The genes encoding the biosynthetic enzymes for hydroxynitrile glucosides in barley were demonstrated to cluster^14^, and in the current study, the identified genomic deletion in cv. Emir was shown to include all known biosynthetic genes, except *CYP71U5* and *CYP79A8*. The latter two genes are located downstream from the core of the gene cluster. CYP79A8 and CYP79A12 both catalyse production of (*E*/*Z*)-3-methylbutyraldoxime when transiently expressed in *Nicotiana benthamiana*^14^. The expression of these genes resulted in necrotic lesions on the leaves. Upon co-expression of the CYP71 gene candidates in *Nicotiana benthamiana*, the necrotic lesions vanished demonstrating that all CYP71 candidates possess catalytic activity. Nevertheless, co-expression of *CYP79A8*, *CYP71U5* and *UGT85F22* did not result in formation of any hydroxynitrile glucosides^14^. We therefore conclude that the deletion harbours all the genes responsible for *in planta* hydroxynitrile glucoside synthesis in barley and is the genetic cause for zero-hydroxynitrile glucoside production in cv. Emir. The SNP analysis of the 227 barley lines supports that zero-hydroxynitrile glucoside accumulators in general all contain a deletion of the core biosynthetic genes, which allows distinguishing between non-producers and producers based on a presence/absence assay of the SNP markers. The need of molecular diagnostics to identify non-producers^18,19,21^ is demonstrated by a commercial screen available since 2005 and used in the Scotch whisky industry, of which a recently released version allows the identification of heterozygotes carrying the null allele (https://www.huttonltd.com/services/molecular-diagnostics). In the cvs. Emir, Pallas, and Mentor, all identified amino acid substitutions resulting from the identified SNPs in the genes encoding the enzymes for hydroxynitrile glucoside biosynthesis were located outside the predicted conserved signature domains of the enzymes (Table 1 and Supplementary Fig. S2). Numerous shared polymorphisms between the cvs. Emir and Mentor in the *CYP71U5* and *CYP79A8* genes imply a common origin of this part of the gene cluster in these two cultivars. The cv. Mentor carries an exclusive P337Q substitution in CYP79A12. This position is outside the predicted substrate recognition sites and heme-binding domain, but an effect of this substitution on the catalytic activity of the key enzyme cannot be excluded. In sorghum, an E145K substitution in the CYP79A1 enzyme was reported to result in increased hydrogen cyanide potential^26^. The P337Q substitution observed in cv. Mentor is missing in the cvs. Bonus and Bowman, which produce medium-levels of hydroxynitrile glucosides (not shown), and in Haruna Nijo, where no data about the compound level are available. It is unclear whether this substitution may have a similar effect on the enzyme activity. Some substitutions unique for either cv. Pallas or cv. Mentor were identified in CYP71C103 and CYP71C113. A substitution in a single cultivar means that the other cultivar shares similarity to cv. Morex. Thus, the substitutions are not conserved in medium- and high-level producers. It is therefore unlikely that those substitutions affect enzyme activity and thereby the total hydroxynitrile glucoside content. Several substitutions were found in CYP71U5. However, as discussed above, CYP71U5 is not involved in hydroxynitrile glucoside biosynthesis *in planta*. None of the substitutions found in the two UGTs were located within the predicted UDP-glycosyltransferase signatures. An effect of those substitutions on the total content is considered to be unlikely.

HPLC-based analysis of the hydroxynitrile glucoside profiles in barley demonstrated the biosynthesis of all five compounds in the cvs. Pallas and Mentor early in plant development (Fig. 2a). This result is in agreement with a previous report where shoot tissue harvested 3 days after seed wetting was found to contain the hydroxynitrile glucosides^13^. In general, biosynthesis of cyanogenic glucosides early in plant development is a common trait among hydroxynitrile glucoside-producing species^4^, indicating a crucial role of these bioactive natural products during germination and establishment of growth. Although substantial variations in the total content were observed between the cvs. Pallas and Mentor, the ratio between the individual hydroxynitrile glucosides was found to remain the same in the two barley cultivars and at all experimental setups. A constant ratio between the five hydroxynitrile glucosides was previously reported in the analysis of 247 spring barley lines including landraces and old and modern cultivars^14^. In addition, conserved ratios were reported from species and cultivars within the genus of *Ribes*, in accessions of *Rhodiola rosea*, and in *L. japonicus*, where the β- and γ-hydroxynitrile glucosides were the dominating types^27^. This is similar in barley, where the α-hydroxynitrile glucoside epiheterodendrin accounts for ~15% of the total content, the β-hydroxynitrile glucoside epidermin for ~22%, and the y-hydroxynitrile glucosides osmaronin, dihydroosmaronin, and sutherlandin for ~63%^14^. At the molecular level, the constant ratio between the α-, β- and γ-hydroxynitrile glucosides may be ascertained by organization of the biosynthetic enzymes within a structurally stable metabolon. In sorghum, the enzymes catalysing biosynthesis of the cyanogenic glucoside dhurrin have been shown to be organized within a dynamic metabolon^28–30^.

Expression analysis of the hydroxynitrile glucoside biosynthetic genes in the cvs. Pallas and Mentor revealed that all genes were significantly higher expressed in the high-level producer cv. Mentor (Fig. 3). The highest upregulation was observed for *CYP79A12* encoding the enzyme carrying out the initial conversion of the parent amino acid into the corresponding E-oxime. Thus, the influx of oximes may be maintained by differential transcriptional regulation of *CYP79A12* in the cvs. Pallas and Mentor. Transcriptional regulation of the CYP79 enzyme was also reported for dhurrin biosynthesis in young sorghum plants^31^ and in developing sorghum grains^32^. The smallest difference in transcript expression between the cultivars was found for *UGT85F22*. However, it is noteworthy that the transcript levels of all CYP71 encoding genes were in general higher than that of the CYP79A12 and UGT85F22 encoding genes, as their amplicons came up at earlier ct values in both cultivars at any time points (data not shown). Our result is in agreement with the observations in developing sorghum grains (17 days after anthesis), where *CYP71E1* gene expression was higher than that of *CYP79A1*^32^. In that study, the gene encoding UGT85B1 showed the highest expression, which is in contrast to our data, where the *CYP79A12* and *UGT85F22* transcripts came up at close ct values at most time points (not shown). In an earlier study, it was suggested that this ratio between the expression of the UGT, CYP71, and CYP79 genes could prevent release of toxic intermediates^33^ like the oxime which causes necrotic lesions^14^.

The concerted up- and down-regulation together with the close proximity of the genes in the cluster implies a coordinated regulation of hydroxynitrile glucoside biosynthetic gene transcription in barley. The (*E*)-3-methylbutanal oxime is the first biosynthetic intermediate and shared precursor of all five compounds. Coordinated regulation of hydroxynitrile glucoside biosynthesis could be executed by negative feedback on the oxime-level as reported from taxiphyllin-producing seaside arrow grass^34,35^. It has previously been shown that CYP79A12, CYP71C113, CYP71L1, and UGT85F22 were able to produce all five barley hydroxynitrile glucosides in barley^14^. The respective genes are in close proximity to each other within the biosynthetic gene cluster on the same strand of barley chromosome 1 H. Hence, a common transcription factor may possibly induce *de novo* hydroxynitrile glucoside biosynthesis. In almond (*Prunus dulcis*), the key event enabling almond domestication was the selection of genotypes harbouring sweet kernels, as wild almond species accumulate the bitter and toxic cyanogenic diglucoside amygdalin. Non-synonymous point mutations in a bHLH transcription factor have recently been documented to obliterate expression of *PdCYP79D16* and *PdCYP71AN24* encoding the two P450s involved in amygdalin synthesis and thereby generate the sweet kernel trait^36^. In addition to transcriptional regulation, biosynthesis could further be controlled at the protein level by metabolon formation as discussed above. Co-expression of fluorescence-tagged fusion proteins of the sorghum CYP79A1 and CYP71E1 for dhurrin biosynthesis in transgenic *Arabidopsis thaliana* and confocal laser microscopy demonstrated that tight interaction of both enzymes is required for dhurrin biosynthesis^37^. It was also shown that the presence of CYP79A1 and CYP71E1 results in a shifted localization of UGT85B1 from the cytosol towards the surface of the ER membrane in biosynthetically active cells^37^. Recently, these results were substantiated by direct isolation of the dhurrin metabolon^28,29^. The presence of an equivalent metabolon in barley is likely and would in addition to CYP79A12 incorporate at least two CYP71 enzymes and a UGT85. The existence of a metabolon in barley is also supported by the observed requirement of the simultaneous presence of CYP71C113 and CYP71L1 in order for the last hydroxylation in the sutherlandin pathway to take place^14^.

The multifunctional properties of cyanogenic glucosides in plants render assessment of their *in planta* functions mottled. Dependent on growth conditions and the prevalence of specific pests or herbivores, the presence or absence of hydroxynitrile glucosides may represent an advantage or disadvantage. In barley, the hydroxynitrile glucosides accumulate in the leaf epidermal cells. This is the site of attack, growth and reproduction of the fungus *Blumeria graminis* f. sp. *hordei*, the causal agent of the economically important disease powdery mildew^13^. In *in vitro* experiments, the cyanogenic glucoside epiheterodendrin increases spore germination frequency and promotes appressoria and appressorial hook formation in *B. graminis*^23^. This could imply that epiheterodendrin is used by the powdery mildew fungus as a recognition factor and following successful infection of epidermal cells as a nutrient source. Similar effects are not observed with the β- and γ-hydroxynitrile glucosides. Absence of hydroxynitrile glucosides might thus give the cultivar increased resistance to barley powdery mildew while rendering it more susceptible to other pests. In general, non-adapted pests and herbivores are assumed to be negatively affected by the presence of hydroxynitrile glucosides. Non-adapted insects may tolerate the presence of hydroxynitrile glucosides in the ingested plant material by keeping the compounds intact for excretion, by an ability to metabolize the compounds, or by avoiding uptake^38,39^. In addition to these approaches, some specialist insects store ingested toxins in special tissues and secrete the toxins as an active immediate response upon predator attack^40–42^. Larvae of *Zygaena filipendulae* (Lepidoptera) secrete viscous hydroxynitrile glucoside-containing droplets from segmentally arranged cavities in their thick cuticle. When ingested by a predator, the droplets may detonate a hydrogen cyanide bomb and glue mandibles and legs of potential predators together and immobilize them. The larvae may reabsorb the defence droplets when the irritation stops^41,42^.

Further studies are required to unravel the fine-tuning of hydroxynitrile glucoside biosynthesis in barley and the physiological role of the individual as well as the co-occurring assemblies of different hydroxynitrile glucosides. The level of ethyl carbamate theoretically remains a concern in whisky production, but is efficiently avoided by strict adherence to the use of barley cultivars that are zero-producers of hydroxynitrile glucosides as a result of the genome deletion presented in this study that eliminates the gene cluster encoding the enzymes catalysing hydroxynitrile glucoside biosynthesis. The loss of the gene cluster may lead to losses of yet unrecognized positive physiological functions of the β- and γ-hydroxynitrile glucosides e.g. as protectants against specific fungal or insect diseases. Our study provides new starting points to investigate the superordinate control mechanisms of hydroxynitrile glucoside biosynthesis in barley and organization of the pathway within a metabolon.

## Materials and Methods

### Plant material

A new seed batch was generated for cv. Emir by selecting grains from plants that were positively tested for complete absence of hydroxynitrile glucosides. Seeds of the barley cvs. Emir, Pallas and Mentor were grown on moist filter paper in transparent boxes at 20 °C and a photoperiod of 13 h (250 µmol m^−2^ s^−1^ photon flux density). Shoot tissue was collected daily in three biological replicates from day 3 to 7 after seed wetting. Each biological replicate was composed of three individual plants, and any given plant was only sampled once. The collected shoot tissue (approx. 1.5 cm) was ground in liquid nitrogen. For RNA isolation, a modified protocol from Yang *et al*.^43^ was used, with the exception that only 900 μl of extraction buffer I were used for the initial step. Each RNA pellet was resuspended in 30 μl nuclease-free water. RNA purity was checked by spectrophotometry and integrity was confirmed through electrophoresis on a 1% agarose gel. Approximately 1 μg RNA of each sample was treated with DNase I, RNase-free (Thermo Fisher Scientific, Waltham, US-MA) following the manufacturer’s recommendation to avoid genomic DNA contamination. Subsequent cDNA synthesis was performed using the iScript^TM^ cDNA synthesis kit (Bio-Rad, Hercules, US-CA). The cDNAs were 10-fold diluted with nuclease-free water to a final concentration of approximately 220 ng/μl. The cDNA purity was checked by spectrophotometry and cDNA integrity was confirmed through PCR and electrophoresis on a 1% agarose gel. The PCR was performed using a specific, intron-spanning primer pair, giving different amplicons for cDNA and genomic DNA. Contamination with genomic DNA could be excluded for all samples. For HPLC-MS analysis, 300 μl of 85% methanol containing 50 μM amygdalin as internal standard were added to a determined portion of the above mentioned ground material and samples were heated at 70 °C for 10 min. After a short cooling step on ice, the extract were 10-fold diluted with deionized water and filtered through a 22-μm Ultrafree-MC Durapore PVDF filter (Merck, Billerica, US-MA) prior to HPLC-MS analysis.

### Bioinformatics, sequencing, and sequence alignments

Updated sequence information for the hydroxynitrile glucoside biosynthesis gene cluster was obtained by blasting the coding sequences of the biosynthetic enzymes described by Knoch *et al*.^14^ against the recent barley genome assembly^24^. Different amplification strategies were required in order to amplify and sequence the biosynthetic genes from the cvs. Emir, Pallas, and Mentor. Genomic DNA was isolated from leaf tissue. For DNA extraction and PCR, either the Extract-N-Amp Plant PCR Kit (Merck) or DNeasy Plant Mini Kit (Qiagen, Hilden, Germany) and Phusion® High-Fidelity PCR Master Mix with HF Buffer (Thermo Fisher Scientific) were used. The primer sequences are shown in Supplementary Table S3, annealing temperatures and amplification strategies used can be found in Supplementary Table S4. The PCR products were purified with the NucleoSpin® Gel and PCR Clean-up Kit (MACHEREY-NAGEL, Düren, Germany) or the NucleoFast® 96 PCR Clean-up Kit (MACHEREY-NAGEL). The purified products were Sanger sequenced by Eurofins (Eurofins Scientific, Ebersberg, Germany) or StarSEQ (StarSEQ GmbH, Mainz, Germany). Sequencing results were analysed using the CLC main workbench software (version 7.7) (Qiagen).

Except for the *CYP71L1* gene, the predicted splicing variants expressed in cv. Morex (http://webblast.ipk-gatersleben.de) were used as references for the different alignments. The transcript used for the *CYP71L1* gene was found on Ensembl Plants (http://plants.ensembl.org; gene ID: HORVU1Hr1G007830.1). For the *CYP79A8* gene, 36 nucleotides were removed in the start of the gene to match the start of an open reading frame. Available sequence information from the cvs. Haruna Nijo, Bowman, and Bonus were included in the alignments. The coding sequences from the cv. Haruna Nijo^44^ were identified by blastn search (https://www.ncbi.nlm.nih.gov/BLAST) of the cv. Morex sequences. Genomic sequences from cv. Bowman^45^ were found by blastn search (http://webblast.ipk-gatersleben.de), and predicted coding sequences were obtained by comparison to the coding sequences from cv. Morex. For *CYP79A12* and *CYP79A8*, the original sequences from cv. Bonus were included (https://www.nih.gov; GenBank FJ593638.1 and FJ455416.1).

### SNP markers analysis

The total hydroxynitrile glucoside contents of 247 barley lines including landraces, old cultivars (1883–1980) and modern cultivars and breeding lines (1981–2013) were previously analysed^14^. 227 of those lines were further genotyped using the 7842 SNP Illumina iSelect array^46^. Using blastn, 13 SNP markers located within or in close proximity to the biosynthetic gene cluster on chromosome 1H were identified, and the SNP patterns for the 227 barley lines were extracted. A summary of the 227 lines can be found in Supplementary Table S5.

### HPLC-MS analysis

Chromatography was performed on an Advance UHPLC system (Bruker, Bremen, Germany). Separation was achieved on a Kinetex 1.7 u XB-C18 column (100 × 2.1 mm, 1.7 µm, 100 Å) (Phenomenex, Torrance, US-CA). 0.05% formic acid (HCOOH) and acetonitrile (MeCN)/0.05% HCOOH were employed as mobile phases A and B, respectively. The elution profile was: 0–0.2 min, 2% B; 0.2–1.8 min, 2–30% B; 1.8–2.5 min, 30–100% B; 2.5–2.8 min, 100% B; 2.8–2.9 min, 100–2% B; 2.9–4.0 min, 2% B. The mobile phase flow rate was 400 µl min^−1^. The column temperature was maintained at 40 °C. The UHPLC was hyphenated to an EVOQ Elite Triple Quad mass spectrometer (Bruker) equipped with an electrospray ion source (ESI) operated in positive ion mode. Instrument parameters were optimized by infusion experiments with chemically synthesized pure standards^14^. The ion spray voltage was maintained at +4500 V in the positive ionization mode. Cone temperature was set to 300 °C and cone gas to 20 psi. Heated probe temperature was set to 175 °C and probe gas flow to 50 psi. Nebulizing gas was set to 60 psi and collision gas to 1.6 mTorr. Nitrogen was used as probe and nebulizing gas and argon as collision gas. Active exhaust was constantly on. Multiple reaction monitoring (MRM) (positive mode) was used to monitor analyte parent ion → product ion transitions. MRMs were chosen based on MRM identification by direct infusion experiments with standards of the hydroxynitrile glucosides. Detailed values for mass transitions can be found in Supplementary Table S6. Both Q1 and Q3 quadrupoles were maintained at unit resolution. Bruker MS Workstation software (version 8.1.2) (Bruker) was used for data acquisition and processing. Linearity in ionization efficiencies was verified by analysing dilution series. Standard curves were performed for all barley hydroxynitrile glucosides by using the chemically synthesized and NMR-verified standards. Injection volume was 1 µl. T-test was used to calculate statistically significant differences.

### Quantitative real-time PCR studies

Gene expression studies were carried out with the transcripts of *CYP79A12*, *CYP71L1*, *CYP71C103*, *CYP71C113*, and *UGT85F22*. Due to high sequence similarity between the *CYP79A12* and *CYP79A8* genes, the primers designed for the *CYP79A12* gene may in addition amplify the *CYP79A8* gene product. However, the *CYP79A8* gene is annotated as non-translating cds and no expression data are available. The *CYP71U5* gene was excluded as it is not expressed in shoot tissue^14^. No functional primers were obtained for the *UGT85F23* gene; thus, it was also excluded. Three reference genes were included in the study: *elongation factor 1 alpha*, *small nucleolar RNA snoR14*, and *CWC15 homolog*. Primer sequence information for *small nucleolar RNA snoR14* was found in Ferdous *et al*.^47^. All other primers were designed using the CLC Main Workbench software. Primer sequences and gene accession numbers are summarized in Supplementary Table S3. Primers were synthesized by Integrated DNA Technologies (IDT, Coralville, US-IA) and specificity was confirmed through agarose gel electrophoresis, melting curve analysis after quantitative real-time PCR, and sequencing of the amplicons by Eurofins (Eurofins Scientific). Samples for quantitative real-time PCR were prepared using the KAPA SYBR® FAST master mix (Merck) and approximately 2 μl template cDNA. The quantitative real-time PCR was conducted using a CFX384 Touch Real-Time PCR Detection System coupled to the CFX Manager software (Bio-Rad). Amplification conditions were 3 min at 95 °C, followed by 40 cycles of 10 s at 95 °C, 30 s at 56 °C, and 1 s at 72 °C. A subsequent melting curve analysis was performed ranging from 60 °C to 95 °C with an increment of 0.5 °C every 5 s. Ct values were extracted from the software and further analysed using the GeNorm algorithm^48^. T-test was used to calculate statistically significant differences.

## Supplementary information

Supplementary data

## Data Availability

The data sets supporting the results of this article are included within the article and its additional files are available upon reasonable request.
