# Supplementary material for: Deletion of biosynthetic genes, specific SNP patterns and differences in transcript accumulation cause variation in hydroxynitrile glucoside content in barley cultivars
Source: Sci Rep. 2019 Apr 5;9:5730. doi: 10.1038/s41598-019-41884-w (PMC6450869; doi:10.1038/s41598-019-41884-w)
Supplement: Supplementary file 1 — Supplementary data [file 41598_2019_41884_MOESM1_ESM.pdf]

Deletion of biosynthetic genes, specific SNP patterns and differences in transcript accumulation cause variation in hydroxynitrile glucoside content in barley cultivars

Marcus Ehlert[1,2,#], Lea Møller Jagd[3,#], Ilka Braumann[3], Christoph Dockter[3], Christoph Crocoll[4], Mohammed Saddik Motawia[1,2], Birger Lindberg Møller[1,2], Michael Foged Lyngkjær[1,2,*]

[1]Plant Biochemistry Laboratory, Department of Plant and Environmental Sciences, University of Copenhagen, Thorvaldsensvej 40, 1871 Frederiksberg C, Copenhagen, Denmark.

[2]VILLUM Research Center for Plant Plasticity, University of Copenhagen, Thorvaldsensvej 40, 1871 Frederiksberg C, Copenhagen, Denmark.

[3]Carlsberg Research Laboratory, J.C. Jacobsens Gade 4, 1799 Copenhagen V, Denmark.

[4]DynaMo Center, Department of Plant and Environmental Sciences, University of Copenhagen, Thorvaldsensvej 40, 1871 Frederiksberg C, Denmark.

[#]These authors contributed equally to the work.

*Corresponding author: Michael Foged Lyngkjær (mlyn@plen.ku.dk)

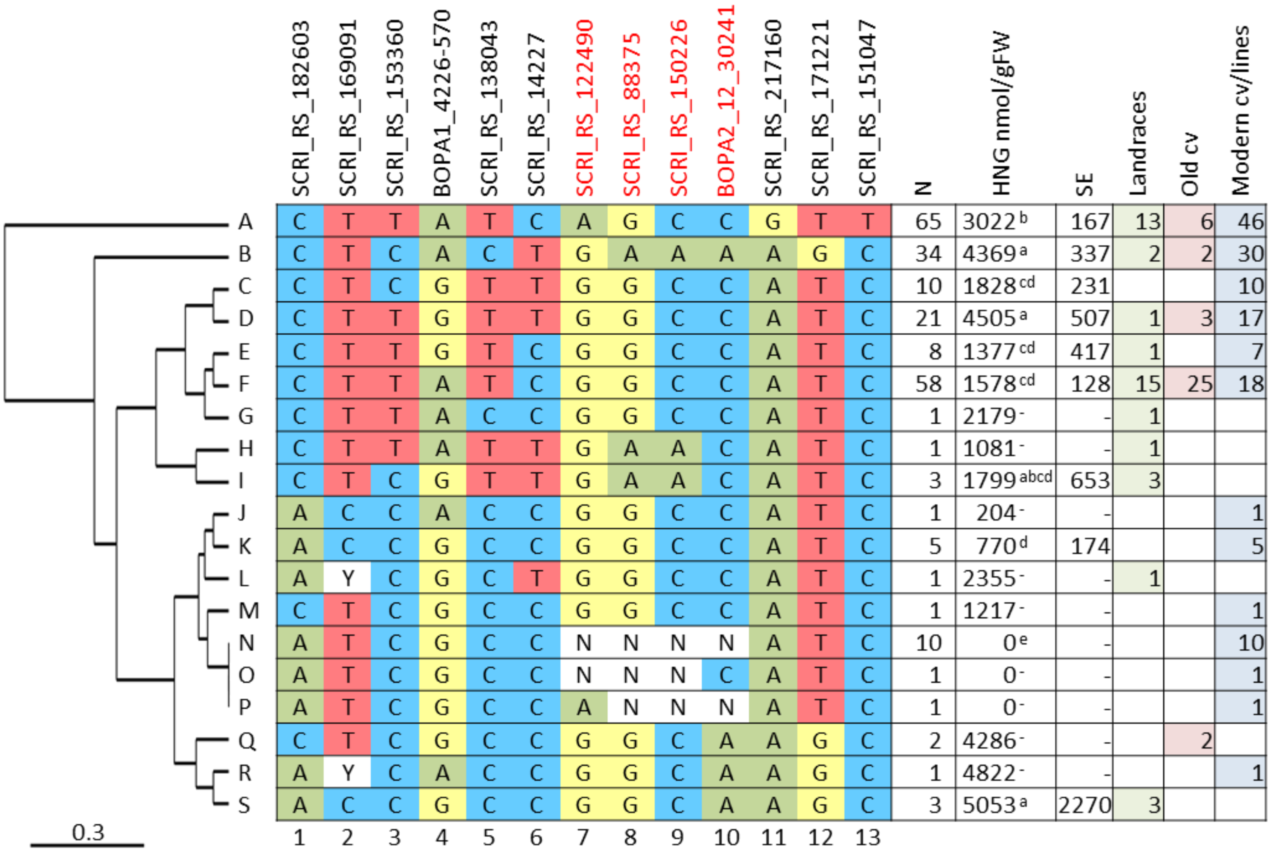

	SCRI_RS_182603	SCRI_RS_169091	SCRI_RS_153360	BOPA1_4226-570	SCRI_RS_138043	SCRI_RS_14227	SCRI_RS_122490	SCRI_RS_88375	SCRI_RS_150226	BOPA2_12_30241	SCRI_RS_217160	SCRI_RS_171221	SCRI_RS_151047	N	HNG nmol/gFW	SE	Landraces	Old cv	Modern cv/lines
A	C	T	T	A	T	C	A	G	C	C	G	T	T	65	3022^{b}	167	13	6	46
B	C	T	C	A	C	T	G	A	A	A	A	G	C	34	4369^{a}	337	2	2	30
C	C	T	C	G	T	T	G	G	C	C	A	T	C	10	1828^{cd}	231			10
D	C	T	T	G	T	T	G	G	C	C	A	T	C	21	4505^{a}	507	1	3	17
E	C	T	T	G	T	C	G	G	C	C	A	T	C	8	1377^{cd}	417	1		7
F	C	T	T	A	T	C	G	G	C	C	A	T	C	58	1578^{cd}	128	15	25	18
G	C	T	T	A	C	C	G	G	C	C	A	T	C	1	2179^{-}	-	1		
H	C	T	T	A	T	T	G	A	A	C	A	T	C	1	1081^{-}	-	1		
I	C	T	C	G	T	T	G	A	A	C	A	T	C	3	1799^{abcd}	653	3		
J	A	C	C	A	C	C	G	G	C	C	A	T	C	1	204^{-}	-			1
K	A	C	C	G	C	C	G	G	C	C	A	T	C	5	770^{d}	174			5
L	A	Y	C	G	C	T	G	G	C	C	A	T	C	1	2355^{-}	-	1		
M	C	T	C	G	C	C	G	G	C	C	A	T	C	1	1217^{-}	-			1
N	A	T	C	G	C	C	N	N	N	N	A	T	C	10	0^{e}	-			10
O	A	T	C	G	C	C	N	N	N	C	A	T	C	1	0^{-}	-			1
P	A	T	C	G	C	C	A	N	N	N	A	T	C	1	0^{-}	-			1
Q	C	T	C	G	C	C	G	G	C	A	A	G	C	2	4286^{-}	-		2	
R	A	Y	C	A	C	C	G	G	C	A	A	G	C	1	4822^{-}	-			1
S	A	C	C	G	C	C	G	G	C	A	A	G	C	3	5053^{a}	2270	3		
	1	2	3	4	5	6	7	8	9	10	11	12	13						

Supplementary Figure S1. SNP variation of 227 barley lines based on 13 SNP markers located within or in close proximity to the hydroxynitrile glucoside biosynthesis gene cluster. Sequence relationship of the different SNP patterns (A-S) identified is listed together with the number (N) of lines sharing the particular SNP pattern, their average total hydroxynitrile glucoside (HNG) content in nmol per gram fresh weight (nmol/gFW) (Knoch *et al.*, 2016), standard error (SE) and distribution in landraces, old cultivars (Old cv), and modern cultivars and breeding lines (Modern cv/lines). Names of SNP markers are given above the columns and the numbers below correspond to their chromosome location in Figure 1. Different small letters next to the average total HNG contents indicate significantly different values according to t-statistic. Only SNP patterns shared by at least 3 barley lines were included in the statistical analysis.

CYP79A12

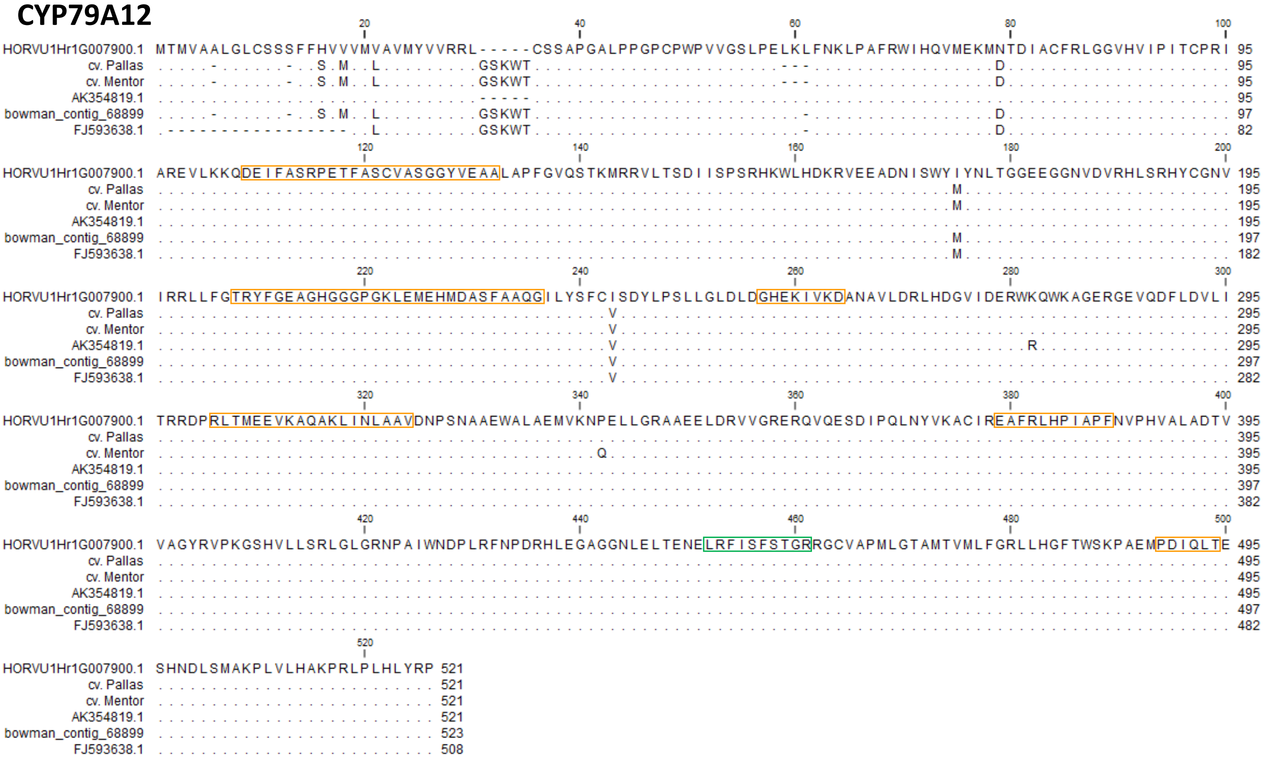

CYP79A8

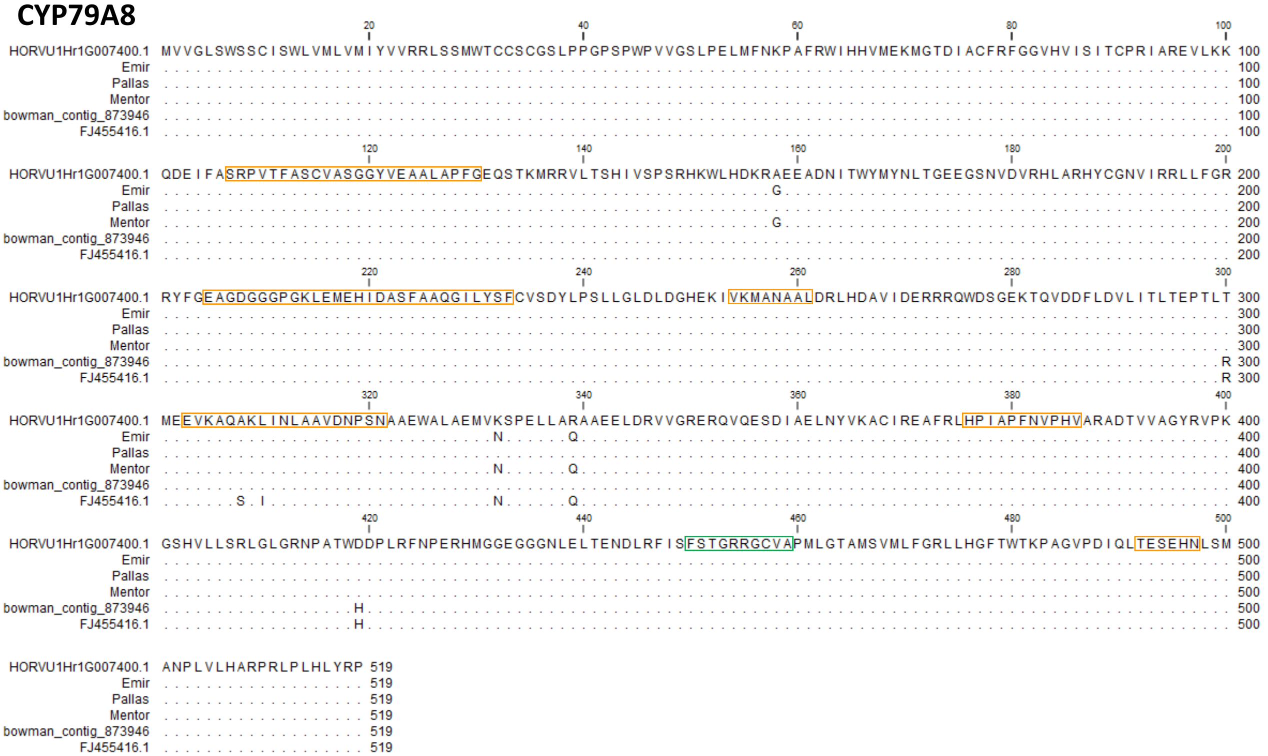

CYP71U5

```
                        20                  40                  60                  80                  100
                         |                   |                   |                   |                   |
HORVU1Hr1G007420.5   MDMDQLSYGSLWVMAVATALFWLILRKAIYVGGKDDTGAKAKLPPGPWNLPVIGSLHHLVVTKLPPHRALLRLSRRHGPLMLVWLGEVPSIVVSSPEAAK 100
Emir                 .................................................................................................... 100
Pallas               .................................................................................................... 100
Mentor               .................................................................................................... 100
bowman_contig_878553 .................................................................................................... 100

                        120                 140                 160                 180                 200
HORVU1Hr1G007420.5   EVLKTNDLVFANRPCGPTMDIVSCGGKGILLAPYGDHWRQMRKVCVVEVLSARQVRRIESIQQAEVARLLESVSAAATGCAVVDVGKALAELSSNIIATA 200
Emir                 .......................................P............................................................ 200
Pallas               .................................................................................................... 200
Mentor               .......................................P............................................................ 200
bowman_contig_878553 .................................................................................................... 200

                        220                 240                 260                 280                 300
HORVU1Hr1G007420.5   VFGGKFPQQEAFLREIDALSVLVGGFSMADLFPSSRLVRWLSSATHDVKRSHARVQRILEDIIQERKEKTSKNGASSVARDNEDLLDVLLRLQRDDTLNF 300
Emir                 .............................................................................A...................... 300
Pallas               .............................................................................A....................S. 300
Mentor               .............................................................................A...................... 300
bowman_contig_878553 .............................................................................A....................S. 300

                        320                 340                 360                 380                 400
HORVU1Hr1G007420.5   PLTSEIIDCVIRDIIGAATETTSSTIEWAMAELVGNPEAMAKAKHEVRERCHGVVASADIGELQYLRMVIKETLRLHPAGMFHRASLEDCQVMGYHIPKG 400
Emir                 .....................................................I.............................................. 400
Pallas               .................................................................................................... 400
Mentor               .....................................................I.............................................. 400
bowman_contig_878553 .................................................................................................... 400

                        420                 440                 460                 480                 500
HORVU1Hr1G007420.5   TAVMINGFAVGTDPAHWGEDAGEFRPERFHDMEMTEYMQMEFVPFGAGRRQCPGTLFATTIMELVLAHLLCHFDWEVPDGQTLDMGEDYGFIVHTRSSLR 500
Emir                 .....................S.............................................N........T...E................... 500
Pallas               .................................................................................................... 500
Mentor               .....................S.............................................N........T...E................... 500
bowman_contig_878553 .................................................................................................... 500

HORVU1Hr1G007420.5   LQASSIVPSC 510
Emir                 .......... 510
Pallas               .......... 510
Mentor               .......... 510
bowman_contig_878553 .......... 510
```

CYP71L1

```
                     20                  40                  60                  80                  100
                      |                   |                   |                   |                   |
HORVU1Hr1G007830.1 MAAASLVLELLRQQWQVTVAILLLPLASFLLTRRRSSNLNGCNESGGLRLPPCPWRLPMVGNLHQIGSLPHRDLARLARRHGPVMMVRLGMVPAVVLSTA 100
cv. Pallas         ..........................................D......................................................... 100
cv. Mentor         ..........................................D......................................................... 100
AK248375.1         ..........................................D......................................................... 100

                     120                 140                 160                 180                 200
HORVU1Hr1G007830.1 AAAEEAFKTNDKDCSSRPLTVGPGKLTYGYKDVVFAPWSDYVREMRKLFIIEMLSARRVKAAYFARETQIERMVAKLEAVGPNPIRIDEHIFTTVDAIVS 200
cv. Pallas         .................................................................................................... 200
cv. Mentor         .................................................................................................... 200
AK248375.1         .................................................................................................... 200

                     220                 240                 260                 280                 300
HORVU1Hr1G007830.1 LFVFGELNAGEQFKGELVDLLNETTDLLTSFTAEDYFPNAAGRLIDRITGMHGRRETLFRKLDSMMEYLLAMYEDPGHKRKADADGSDLVQEVVDLMKRP 300
cv. Pallas         .................................................................................................... 300
cv. Mentor         .................................................................................................... 300
AK248375.1         .................................................................................................... 300

                     320                 340                 360                 380                 400
HORVU1Hr1G007830.1 PAKGMITFTRDHAKSILFDTFMAATDTSSISSYWVMTELIRHPRVLHKAQAEVRAAAGGAPQVRISDMPKLKYLRMVLSETFRMHPPATMLVPRETMRPI 400
cv. Pallas         .................................................................................................... 400
cv. Mentor         .................................................................................................... 400
AK248375.1         .................................................................................................... 400

                     420                 440                 460                 480                 500
HORVU1Hr1G007830.1 RLGGYDIPANTMLMVNAWAIGRDPASWKDPEVFYPERFEELDVDFNGGHYELLPFGAGRRICPGLAMGVANTEFILANLLYCFNWALPQGMRSEDVGVEE 500
cv. Pallas         .................................................................................................... 500
cv. Mentor         .................................................................................................... 500
AK248375.1         .................................................................................................... 500

                     520
HORVU1Hr1G007830.1 FGGLTFRKKKPLVLPTRYYPDKEEK 526
cv. Pallas         ......................... 526
cv. Mentor         ......................... 526
AK248375.1         ......................... 526
```

CYP71C103

```
                                       20                  40                  60                  80                 100
HORVU1Hr1G007840.2   MEVPHADAALLVLLASILLLMARRRFGLGNAAARAREEALNKLPSPGWRLPVIGHLHQVGPLPHVSLRHLAAEHGRDGLMLVRLGAVPTLVVSTPAAAQA 100
cv. Pallas           .................................................................................................... 100
cv. Mentor           .................................................................................................... 100
AK360412.1           .................................................................................................... 100
bowman_contig_221370 .................................................................................................... 100
                                      120                 140                 160                 180                 200
HORVU1Hr1G007840.2   VLRTHDHVFASRPHSPVAHILFYGSADVVFAPYGHHWRQVKKISTTHLLTARKVHSYRHARQHEVNLVLAKVRDAMRAGVALDMSELLNAFVFDIVCHAV 200
cv. Pallas           .................................................................................................... 200
cv. Mentor           ....................................................................................T............... 200
AK360412.1           .................................................................................................... 200
bowman_contig_221370 .................................................................................................... 200
                                      220                 240                 260                 280                 300
HORVU1Hr1G007840.2   AGNSFRERGLNKHFRELVEANASLIGGFNLEDHFPALVKLEIFRKIVCAKARRVNNKWDDLLDRLIDEHATPPALDEDRDFIHVLLSVQQEYNLTRDHIK 300
cv. Pallas           .................................................................................................... 300
cv. Mentor           .................................................................................................... 300
AK360412.1           .................................................................................................... 300
bowman_contig_221370 .................................................................................................... 300
                                      320                 340                 360                 380                 400
HORVU1Hr1G007840.2   AQLLIMFEAGTDTSTIVLEYAMVRLMQNPRVMAMLQAEVRSTIPKGKDTVTQDDLHGLPYLKAVIKETLRLHMPGPLMVPHLSMDECIINGYTIPSGTRT 400
cv. Pallas           .......................................................................................T............ 400
cv. Mentor           ....................................................E............................................... 400
AK360412.1           .................................................................................................... 400
bowman_contig_221370 .................................................................................................... 400
                                      420                 440                 460                 480                 500
HORVU1Hr1G007840.2   FINTYAIQRDPSNWESPEEFMPERFMEGGSAAAMDYKGNDFQYFPFGSGRRICPGINFATATIQLMLTNLMYHFDWKLPPESEEEGINMTETFGLTVHRK 500
cv. Pallas           .................................................................................................... 500
cv. Mentor           ...........T......................................................................A................. 500
AK360412.1           .................................................................................................... 500
bowman_contig_221370 .................................................................................................... 500

HORVU1Hr1G007840.2   EKLLLVPLVPQN 512
cv. Pallas           ............ 512
cv. Mentor           ............ 512
AK360412.1           ............ 512
bowman_contig_221370 ............ 512
```

CYP71C113

```
                                       20                  40                  60                  80                 100
HORVU1Hr1G007790.1   MGEVPSDELLLVLVSAALLVTILVMVRRSGSAARVREELLRKLPSPGGWLPVIGHLHMVGSQPHVSLGDLAAKHSRDGLMLLRLGSVPTLIVTSSNAARA 100
cv. Pallas           .......G.....................R...................................................................... 100
cv. Mentor           ........................T...............C........................................................... 100
AK355475.1           .................................................................................................... 100
bowman_contig_846052 .................................................................................................... 100
                                      120                 140                 160                 180                 200
HORVU1Hr1G007790.1   VLRTHDDVFASRPHNPATDIIFYGPSDIAFCPYGDHWRQVKKIAMTHLLTANKVRSYRQAREEEACLVVAKLRDAMAAGAALDLGELLSAFSTNIVGHAV 200
cv. Pallas           .................................................................................................... 200
cv. Mentor           .................................................................................................... 200
AK355475.1           .................................................................................................... 200
bowman_contig_846052 .................................................................................................... 200
                                      220                 240                 260                 280                 300
HORVU1Hr1G007790.1   CGKSFRQKGHEKLFRELVETNSLLIGGFNVGDYFPELLKLDIIRWMVCGRARRVNKMWDDLLESLIHEHESKPAPALGDDTDYIHDLLSIQKEYNLTREQ 300
cv. Pallas           .................................................................................................... 300
cv. Mentor           .................................................................................................... 300
AK355475.1           .................................................................................................... 300
bowman_contig_846052 ......................F............................................................................. 300
                                      320                 340                 360                 380                 400
HORVU1Hr1G007790.1   VKAQLVIMFGAGTDTSYIVMEYAMARLMQNPDLMTKLQAEVRSSIPKGKHMVTEDDLNHLAYLKAVIKETLRLHMPAPLLVPHLAMADCVINGYTIPSGT 400
cv. Pallas           .........................................................................................I.......... 400
cv. Mentor           .................................................................................................... 400
AK355475.1           ....................................................I............................................... 400
bowman_contig_846052 .................................................................................................... 400
                                      420                 440                 460                 480                 500
HORVU1Hr1G007790.1   RVIVNSRAIARDPSSWESAEEFLPERFMQGGSAAAMDYKGNGFLYLPFGTGRRICPGINFAIAAIEIMLANLVYHFDWKLPPGSAERGISMTESFGLTVH 500
cv. Pallas           .................................................................................................... 500
cv. Mentor           .................................................................................................... 500
AK355475.1           .................................................................................................... 500
bowman_contig_846052 .................................................................................................... 500

HORVU1Hr1G007790.1   RKDKLLLVPLVPQDYET 517
cv. Pallas           ................. 517
cv. Mentor           ................. 517
AK355475.1           ................. 517
bowman_contig_846052 ................. 517
```

UGT85F22

```
HORVU1Hr1G007810.3   MEMERKPHAVLVPLPQQGHIAPMLKLAKLLHCKAGFHITFVHTEYNQRRLVRSHGPGALAGVPGFRFATIPDGLPPSDADASQDPASICYSTMTTCLPHF 100
cv. Pallas           .................................................................................................... 100
cv. Mentor           .................................................................................................... 100
AK252107.1           ...........................................................T........................................ 100
bowman_contig_126326 .................................................................................................... 100

HORVU1Hr1G007810.3   KKLLQELNATAGMPPVTCVVADNIMSFTLDAAAEVGVPCALFFTASACGYVGYRNFRFLMDKGIAPLKDEAQLTNGYLDTPVPDAPGMSQHMRLRDFPSF 200
cv. Pallas           ....H............................................................................................... 200
cv. Mentor           ....H............................................................................................... 200
AK252107.1           .........P..................V....................................................................... 200
bowman_contig_126326 .................................................................................................... 200

HORVU1Hr1G007810.3   ICTTDRGDVMLNFNLHEVERSGRAAAIIINTLDELEQASLDAMRAILPPPCPVYTIGPLHLLAQGQGQGQVLLPEIPEVLWKADGSCLEWLDGREPGSVV 300
cv. Pallas           ..........................V......................T.................................................. 300
cv. Mentor           ..I.......................V......................T.................................................. 300
AK252107.1           ..........................V......................T.................................................. 300
bowman_contig_126326 .................................................................................................... 300

HORVU1Hr1G007810.3   YVNFGSITTMSGEELVEFAWGLANCGHPFLWIVRKDLLAAKKDDDAAMQLPAEFLQATKGRCLLTSWCDQEAVLQHPALGVFLTHCGWNSALVAISAGVP 400
cv. Pallas           .................................................................................................... 400
cv. Mentor           .................................................................................................... 400
AK252107.1           ......L...............................................R............................................. 400
bowman_contig_126326 .................................................................................................... 400

HORVU1Hr1G007810.3   MLGWPFFAEQQTNCRYASVEWGVGMEVGDNVRRQVVEARIREAMEGDGGNKLKRKAAEWKEICARAAPARSMANLHSLVKDVLMQPPPKTLDSSSSSCLE 500
cv. Pallas           ............................................G....................G.................................. 500
cv. Mentor           ................................................R.............................I..................... 500
AK252107.1           ............................................G....................................................... 500
bowman_contig_126326 .................................................................................................... 500

HORVU1Hr1G007810.3   YEWNVPAHLL 510
cv. Pallas           .......... 510
cv. Mentor           .......... 510
AK252107.1           .......... 510
bowman_contig_126326 .......... 510
```

UGT85F23

```
HORVU1Hr1G007720.1   METKKPHAVCVPLPTQGHVTPLLMLAKLLHLKGFHITFVHTEYNHRRLVRSRGPEAVEGLPDFRFATIPDGLPPSDADATQDPASICYSTMTTCLPHLKK 100
cv. Pallas           .................................................................................................... 100
cv. Mentor           .................................................................................................... 100
AK250769.1           .................................................................................................... 100
bowman_contig_155550 .................................................................................................... 100

HORVU1Hr1G007720.1   LLRELNGTGAAGVPPVTCVVADNVTSFSVDAAAELGVPCALFWTASAAGYMGYCNFRFLMDKGIAPLKDEAQRRNGYLETPVPDAPGMSQRMRLRDFPSF 200
cv. Pallas           .................................................................................................... 200
cv. Mentor           .................................................................................................... 200
AK250769.1           ...............................................................................A.................... 200
bowman_contig_155550 .................................................................................................... 200

HORVU1Hr1G007720.1   ICTTDRGDVMQNFLLHEVERTGRAAAVIVNTLDELERPALDAMRAVLPPTCRVYTIGPLHLLQQEQLLPGIRASLWREDRSCLEWLDGREPGSVVYVNFG 300
cv. Pallas           .................................................................................................... 300
cv. Mentor           .................................................................................................... 300
AK250769.1           .................................................................................................... 300
bowman_contig_155550 .................................................................................................... 300

HORVU1Hr1G007720.1   SITTMSGEELVEFAWGLANSGHPFLWIVRKDLLAAKKDDDAAVRLPPEFVEATKGRCLLASWCEQEAVLQHPALGVFLTHCGWNSTFVAISAGLPMLCWP 400
cv. Pallas           .................................................................................................... 400
cv. Mentor           .................................................................................................... 400
AK250769.1           .................................................................................................... 400
bowman_contig_155550 .................................................................................................... 400

HORVU1Hr1G007720.1   FFAEQQTNSRYACLDDEWGVGLEVGDEVRREVVESRIRAAMGGDDDGGNRLRRKAAEWKEMSARAAAGRSMANLDSLINDVLMQPPPKAIPDSSSPCLE 500
cv. Pallas           ................................................................................................S... 500
cv. Mentor           ................................................................................................S... 500
AK250769.1           ....................E...........................................................................S... 500
bowman_contig_155550 .................................................................................................... 500

HORVU1Hr1G007720.1   YEWNTSGLSL 510
cv. Pallas           .......... 510
cv. Mentor           .......... 510
AK250769.1           .......... 510
bowman_contig_155550 .......... 510
```

Supplementary Figure S2. Alignment of sequenced biosynthetic genes from the cvs. Emir, Pallas, and Mentor together with the available sequence data from cv. Morex and, if available, from the cvs. Haruna Nijo (AK), Bowman (bowman_contig), and Bonus (FJ). Gene sequences were translated to amino acid sequences. Numbers above the sequences refer to the position in the alignment; numbers next to sequences refer to the position of the last amino acid in that row within the protein. Dots indicate matching amino acids, hyphen indicates missing amino acids. Orange label shows predicted substrate recognition sites (in the order from 1 to 6), green label shows predicted cytochrome P450 cysteine heme-iron ligand signatures, blue label shows predicted UDP-glycosyltransferase signatures.

Supplementary Table S3. Primer sequence information for sequencing of the barley hydroxynitrile glucoside biosynthetic genes and for quantitative real-time PCR studies.

Sequencing primers:

Primer	Sequence
LMJ5	TATAAATGAGGGCTAGCTGG
LMJ6	ATCACGGAGCTGTTTGATGG
LMJ7	TCTGTGAGCGTGATGAGG
LMJ8	GATTGTCAAGATGGCCAACG
LMJ10	GCAGGTCCGGTTATTATTAAC
LMJ11	CTGATGCAGAAGGAGTAGAG
LMJ12	TACATTTACAACCTCACCGG
LMJ13	CCCAACTCAACTACGTCAAG
LMJ14	GTAGTTAGATGGTCACTTGC
LMJ15	TCAACAATCAATACTTCACTTGTGA
LMJ16	TGAAGATTTCCAGCTTCACC
LMJ17	TTCGTCTTCGACATCGTGTG
LMJ19	AGAGTGAACAGAACACAAGC
LMJ20	TCCAATCTTGTGTGCACAAC
LMJ21	ACATCTTGTTCACTCTCCTG
LMJ22	CTTCTCCACCAACATCGTG
LMJ23	ACATAGTGATGGAATACGCC
LMJ24	TATTTACCAGTAGTGGTGCG
LMJ25	ACTACTTCTCCTCCTTGTC
LMJ26	AAGGTGATCATGCCCTTAG
LMJ27	GCTGAGCATCTCGATGATG
LMJ28	GTTGCAGCCATGAACGTG
LMJ30	CACCTCGTGGTCACAAAG
LMJ31	CCATAGCAGGTTAATTCTCC
LMJ32	ATCATCGACTGTGTTATCCG
LMJ33	CTATCGTGGAAGAAGTCGTC
LMJ34	AGATAGACTACGACAGATCC
LMJ35	GCATGTCATGAACCTAGC
LMJ36	GCAGGTTGAAGTTAAGCATC
LMJ37	TGACGAACGGGTACCTC
LMJ38	CCAAGAAGGACGACGATG
LMJ39	TTAATCAGCAAGCTAGCACG
LMJ40	GGGAAGGAAATTAAACATGCC
LMJ41	AAGAAGTTCTGCATCACGTC
LMJ42	GAATGGATACCTGGAGACG
LMJ43	CGTCTTCCTCACACACTG
LMJ78	ATGTACCACGAGATGTTG
LMJ79	GAAGCAGGACGAGATCTT
LMJ80	GCCTGCTCCTTCCAGATG
LMJ81	TTGATGAAAGTGCGCGTC
LMJ82	GTCTGCCCTACCTAAAGG
LMJ83	GCCAGATGGTTAAGATCG
LMJ84	TCAAGACCAACGACAAGG
LMJ85	TGATGGAGTATCTGCTGGC

LMJ86	ATAAACTGGAAAGGAGGG
LMJ87	AACGTACTAGGTGAAAGC
LMJ88	GCTGGCGGTAAAGAAGAG
LMJ89	ATCTGCTACTCCACAATGAC
LMJ90	CAGTGCGTGAGGAAGAC
LMJ91	TCGCGTCGAACCTCATC
LMJ92	GTTGAGTAAATAAGGGCAGT
LMJ96	CCGAGCTACCTGCTTACA
LMJ105	ATTGCATGTACGTTGGTTG
LMJ108	GATTTCAGGACACGTTCA
LMJ109	CTTCGTCAATTAGCTCGT

Quantitative real-time PCR primer sequences:

Gene name	Accession number		Primer sequence 5' → 3'
CYP79A12	HORVU1Hr1G007900	Forward	TGCGGCAACGTCATAC
		Reverse	GCAGAAGGAGTAGAGGAT
CYP71L1	HORVU1Hr1G007830	Forward	AAGGATGGTGGCAAAACTCG
		Reverse	CGTCTCGTTCAGCAAGTCCAC
CYP71C103	HORVU1Hr1G007840	Forward	TCATCAACACCTATGCTATCC
		Reverse	AGTCGAAGTGGTACATGAG
CYP71C113	HORVU1Hr1G007790	Forward	AAACACATGGTCACAGAGGA
		Reverse	CTATTGACGATGACGCGC
UGT85F22	HORVU1Hr1G007810	Forward	TGACTTCCCCTCCTTCAT
		Reverse	GCGGGCCAATGGTGTAAA
elongation factor 1 alpha	HORVU5Hr1G100200	Forward	ACCCTGACAAGGTTCCCTTC
		Reverse	ACCAGTCAAGGTTGGTGGAC
small nucleolar RNA snoR14	HORVU3Hr1G014770	Forward	GATGTTTATGTATGATAGTCTGTC
		Reverse	GTCGGGATGTATGCGTGTC
CWC15 homolog	HORVU7Hr1G001880	Forward	AGCTGAGGACAGGCTTAGA
		Reverse	ACGAGCCTGGTTCTTGAATAC

Supplementary Table S4. PCR information for sequencing of barley hydroxynitrile glucoside biosynthesis genes.

CYP71C103	Emir		Pallas		Mentor	
Primers	PCR	Sequencing	PCR	Sequencing	PCR	Sequencing
LMJ14+LMJ16	redex, 56°C	No product	redex, 56°C	LMJ14, LMJ16	redex, 56°C	LMJ14, LMJ16
LMJ17+LMJ81	redex, 56°C	No product	redex, 56°C	LMJ17, LMJ81	redex, 56°C, phusion, 56°C	LMJ17, LMJ81
LMJ17+LMJ15					phusion, 56°C	LMJ17, LMJ82, LMJ81
LMJ82+LMJ15	redex, 56°C	No product	redex, 56°C	LMJ82, LMJ15	phusion, 56°C	LMJ82

CYP71C113	Emir		Pallas		Mentor	
Primers	PCR	Sequencing	PCR	Sequencing	PCR	Sequencing
LMJ19+LMJ21	redex, 56°C	No product	redex, 56°C	LMJ19, LMJ21	redex, 56°C	LMJ19, LMJ21
LMJ22+LMJ83	redex, 56°C	No product	redex, 56°C	LMJ22, LMJ83	redex, 56°C	LMJ22, LMJ83
LMJ23+LMJ20	redex, 56°C	No product	redex, 56°C	LMJ23, LMJ20	redex, 56°C	LMJ23, LMJ20

CYP71L1	Emir		Pallas		Mentor	
Primers	PCR	Sequencing	PCR	Sequencing	PCR	Sequencing
LMJ24+LMJ27	redex, 56°C	No product	redex, 56 C	LMJ24, LMJ27	redex, 56°C	LMJ24, LMJ27
LMJ84+LMJ26	redex, 56°C	No product	redex, 56 C	LMJ84, LMJ26	redex, 56°C	LMJ84, LMJ26
LMJ85+LMJ28	redex, 56°C	No product	redex, 56 C	LMJ85, LMJ28	redex, 56°C, phusion 56°C	LMJ85, LMJ28
LMJ86+LMJ87	redex, 56°C	No product	redex, 56 C	LMJ86, LMJ87		
LMJ85+LMJ87	redex, 56°C	No product			phusion 56°C	LMJ85, LMJ28, LMJ87
LMJ87+LMJ108	phusion, 53°C	No product			phusion, 53°C	LMJ25, LMJ108
LMJ87+LMJ109	phusion, 53°C	No product			phusion, 53°C	LMJ87, LMJ109

CYP71U5	Emir		Pallas		Mentor	
Primers	PCR	Sequencing	PCR	Sequencing	PCR	Sequencing
LMJ30+LMJ31 LMJ32+LMJ33	redex, 58°C	LMJ30, LMJ31, LMJ32, LMJ33	redex, 58°C	LMJ30, LMJ31, LMJ32, LMJ33	redex, 58°C	LMJ30, LMJ31, LMJ32, LMJ33

UGT85F23	Emir		Pallas		Mentor	
Primers	PCR	Sequencing	PCR	Sequencing	PCR	Sequencing
LMJ39+LMJ40	redex, 58°C	No product			redex, 58°C	LMJ39, LMJ40, LMJ41
LMJ39+LMJ41	redex, 56°C	No product	redex, 56°C	LMJ39, LMJ41		
LMJ42+LMJ91	redex, 56°C	No product	redex, 56°C	LMJ42, LMJ91	redex, 56°C	LMJ42
LMJ40+LMJ43	redex, 56°C	No product	redex, 56°C	LMJ40, LMJ43	redex, 56°C	LMJ40, LMJ43

UGT85F22	Emir		Pallas		Mentor	
Primers	PCR	Sequencing	PCR	Sequencing	PCR	Sequencing
LMJ34+LMJ88	redex, 56°C	No product	redex, 56°C	LMJ34, LMJ88	redex, 56°C	LMJ34, LMJ88
LMJ36+LMJ89	redex, 56°C	No product	redex, 56°C	LMJ36, LMJ89	redex, 56°C	LMJ36, LMJ89
LMJ37+LMJ90	redex, 56°C	No product	redex, 56°C	LMJ37, LMJ90	redex, 56°C	LMJ37, LMJ90
LMJ35+LMJ37	redex, 56°C, phusion, 56°C	No product	phusion, 56°C	LMJ37	phusion, 56°C	LMJ35, LMJ37, LMJ38
LMJ38+LMJ35	redex, 56°C	No product	redex, 56°C	LMJ35, LMJ38	redex, 56°C	LMJ35

CYP79A8	Emir		Pallas		Mentor	
Primers	PCR	Sequencing	PCR	Sequencing	PCR	Sequencing
LMJ5+LMJ7	redex, 58°C	LMJ5, LMJ7	redex, 58°C	LMJ5, LMJ7	redex, 58°C	LMJ5, LMJ7
LMJ96+LMJ7			phusion, 56°C, redex, 58°C	LMJ96, LMJ7		
LMJ6+LMJ8	redex, 58°C	LMJ6, LMJ8	redex, 58°C	LMJ6, LMJ8	redex, 58°C	LMJ6, LMJ8

CYP79A12	Emir		Pallas		Mentor	
Primers	PCR	Sequencing	PCR	Sequencing	PCR	Sequencing
LMJ92+11			phusion, 56°C	LMJ92		
LMJ92+78			redex, 56°C	LMJ10, LMJ13	redex, 56°C	LMJ78, LMJ92
LMJ79+LMJ105			redex, 56°C	LMJ12, LMJ105	redex, 56°C	LMJ12, LMJ105
LMJ92+LMJ105			redex, 56°C	LMJ11, LMJ92, LMJ105, LMJ12	redex, 56°C	LM11, LMJ105, LMJ12
LMJ11+LMJ79					redex, 56°C	LMJ11, LMJ79
LMJ10+LMJ107			redex, 55 C	LMJ80	redex, 55 C	LMJ80
LMJ10+LMJ13			redex, 56°C	LMJ10, LMJ13	redex, 56°C	LMJ10, LMJ13

Supplementary Table S5. Hydroxynitrile glucoside contents in 227 barley lines.

	Name	Total HNG	Type	Year of release or first date in the Danish national field trials	Row
HNG1	Breeding line 01	0,00	bl		
HNG2	Hydrogen	0,00	cv	2000	2
HNG3	Helium	0,00	cv	2001	2
HNG4	Breeding line 02	3,32	bl		
HNG5	Breeding line 03	4,19	bl	2011	
HNG6	Breeding line 04	4,46	bl		
HNG7	Breeding line 05	4,62	bl		
HNG8	Breeding line 06	12,33	bl		
HNG9	Breeding line 07	16,46	bl		
HNG10	Breeding line 08	27,50	bl		
HNG11	Breeding line 09	39,05	bl		
HNG12	Breeding line 10	43,81	bl		
HNG13	Brio	190,91	cv	1924	6
HNG14	Breeding line 11	204,13	bl		
HNG15	Breeding line 12	222,17	bl		
HNG16	Breeding line 13	249,00	bl		
HNG17	Karin	258,74	cv	1984	2
HNG18	Breeding line 14	260,41	bl		
HNG19	Bjørne	263,01	lr		6
HNG20	Bryssel KVL 28	271,68	lr		6
HNG21	Breeding line 15	273,19	bl		
HNG22	Lavrans	280,50	cv	1999	2
HNG23	Dønnes	315,16	lr		4
HNG24	Breeding line 16	318,24	bl		
HNG25	Fløya	337,68	cv	1939	6
HNG26	Sarkalahti ME0103	354,56	lr		4
HNG27	Agneta	370,82	cv	1978	2
HNG28	Pirkka	383,45	cv	1952	6
HNG29	Luusua EH0401	413,33	lr		6
HNG30	Breeding line 17	421,21	bl	2012	
HNG31	Jotun	469,18	cv	1930	6
HNG32	Breeding line 19	498,04	bl		
HNG33	Propino	538,55	cv	2009	2
HNG34	Solenbyg	579,69	lr		6
HNG35	Fræg	590,28	cv	1948	6
HNG36	Breeding line 20	645,20	bl		
HNG37	Breeding line 21	741,74	bl	2013	
HNG38	Breeding line 22	773,25	bl		
HNG39	Jadar II	801,04	cv	1947	6
HNG40	Bor 2	802,69	cv	2011	2
HNG41	Langaks	812,72	lr		4

HNG42	Juli Abed	928,41	cv	1909	6
HNG43	Elmeri	929,55	cv	2009	2
HNG44	Kilpau ME0201	935,94	lr		6
HNG45	Åsa	978,86	cv	1949	6
HNG46	Breeding line 24	991,51	bl	2012	
HNG47	Breeding line 25	991,56	bl		
HNG48	Arve	1.001,97	cv	1990	2
HNG49	Breeding line 26	1.010,07	bl		
HNG50	Oppdal	1.081,43	lr		6
HNG51	Stjernebyg fra Færøerne	1.199,51	lr		6
HNG52	SJ 095045	1.216,58	cv	2011	2
HNG53	Breeding line 27	1.220,95	bl		
HNG54	Breeding line 28	1.224,51	bl	2012	
HNG55	Chapeau	1.271,54	cv	2010	2
HNG56	Bor 1	1.333,97	cv	2011	2
HNG57	Breeding line 29	1.348,63	bl		
HNG58	Pallas	1.362,11	cv	1958	6
HNG59	Ljubljana KVL 15	1.377,94	lr		2;4
HNG60	Breeding line 30	1.409,69	bl		
HNG61	Ylenjoki AP0301	1.410,26	lr	0	6
HNG62	Birgitta	1.440,44	cv	1963	2
HNG63	Breeding line 31	1.443,05	bl		
HNG64	Breeding line 32	1.461,65	bl		
HNG65	Junkkari	1.475,20	lr		6
HNG66	Breeding line 33	1.491,28	bl		
HNG67	Breeding line 35	1.546,92	bl		
HNG68	Punto	1.558,13	cv	1995	2
HNG69	Breeding line 36	1.581,92	bl		
HNG70	Hannuksela	1.616,88	lr		6
HNG71	Gaute	1.645,41	cv	2000	2
HNG72	Breeding line 37	1.654,47	bl	2012	
HNG73	Breeding line 38	1.722,67	bl		
HNG74	Lynderupgaard	1.742,03	lr		6
HNG75	Nordslesvigsk Kæmpe	1.759,81	lr		6
HNG76	Vilmorin KVL 126	1.794,12	lr		6
HNG77	Etu	1.804,66	cv	1970	2
HNG78	Breeding line 39	1.809,55	bl		
HNG79	Breeding line 40	1.827,55	bl		
HNG80	Eva	1.844,98	cv	1973	2
HNG81	Breeding line 41	1.847,63	bl		
HNG82	Breeding line 42	1.864,15	bl		
HNG83	Breeding line 43	1.878,06	bl		
HNG84	Bor 3	1.895,61	cv	2011	2
HNG85	Breeding line 44	1.896,32	bl		
HNG86	Breeding line 45	1.916,51	bl		
HNG87	Simba	1.923,52	cv	2002	2
HNG88	Nord	1.976,10	cv	1988	2
HNG89	Odin	1.984,30	cv	1981	2
HNG90	Breeding line 46	2.016,83	bl		
HNG91	Breeding line 47	2.027,64	bl		

HNG92	Caruso	2.046,17	cv	1991	2
HNG93	Piikkiönohra	2.100,33	cv	1922	6
HNG94	Breeding line 48	2.115,37	bl		
HNG95	Brage	2.122,85	cv	2010	2
HNG96	Alabama	2.140,05	cv	1999	2
HNG97	Breeding line 49	2.140,82	bl		
HNG98	Breeding line 50	2.147,60	bl		
HNG99	Denso Abed	2.176,19	cv	1965	6
HNG100	Griechische KVL 56	2.179,31	lr		6
HNG101	Breeding line 51	2.205,70	bl		
HNG102	Breeding line 52	2.258,54	bl		
HNG103	Zita	2.281,21	cv	1974	2
HNG104	Anita Högsby-korn	2.286,50	lr		2
HNG105	Breeding line 53	2.302,09	bl	2011	
HNG106	Lysimax	2.341,75	cv	1994	2
HNG107	Breeding line 54	2.342,27	bl		
HNG108	Nue Grosse	2.354,56	lr		2
HNG109	Breeding line 55	2.378,87	bl		
HNG110	Carlsberg	2.383,58	cv	1946	6
HNG111	Stange	2.387,74	cv	1978	6
HNG112	Evergreen	2.428,24	cv	2010	2
HNG113	Breeding line 56	2.429,52	bl		
HNG114	Acclaim	2.440,46	cv	2012	2
HNG115	Breeding line 57	2.449,81	bl		
HNG116	Breeding line 58	2.458,80	bl		
HNG117	Breeding line 59	2.468,42	bl		
HNG118	Breeding line 60	2.470,45	bl		
HNG119	Breeding line 61	2.473,91	bl		
HNG120	Karri	2.475,34	cv	1967	6
HNG121	Breeding line 62	2.489,49	bl		
HNG122	Landora	2.505,71	cv	2000	2
HNG123	Rauto	2.514,32	lr		2;6
HNG124	Fairytale	2.520,62	cv	2006	6
HNG125	Breeding line 63	2.524,28	bl		
HNG126	Hannchen	2.556,51	cv	1902	2
HNG127	Breeding line 64	2.581,54	bl		
HNG128	Breeding line 65	2.609,84	bl		
HNG129	Simba	2.612,29	cv	2002	2
HNG130	Gunnar	2.615,77	cv	1981	2
HNG131	Mari	2628,06	cv	1960	6
HNG132	Rehakka-65	2.633,08	lr		2
HNG133	NOS 17009-53	2.636,74	cv	2011	2
HNG134	Iron	2.663,19	cv	2007	2
HNG135	Breeding line 66	2.671,08	bl		
HNG136	Breeding line 67	2.678,31	bl		
HNG137	Rex Abed	2.691,45	cv	1913	6
HNG138	Breeding line 68	2.709,15	bl		
HNG139	Breeding line 69	2.709,85	bl		
HNG140	Breeding line 70	2.714,30	bl		
HNG141	Breeding line 71	2.755,14	bl		

HNG142	Columbus	2.760,81	cv	2009	6
HNG143	Cicero	2.780,68	cv	1999	2
HNG144	Breeding line 72	2.824,30	bl	2013	
HNG145	Brazil	2.828,48	cv	2000	2
HNG146	Breeding line 73	2.834,06	bl		
HNG147	Evergreen	2.837,32	cv	2010	2
HNG148	Breeding line 74	2.851,90	bl		
HNG149	Prominant	2.874,77	cv	1999	2
HNG150	Breeding line 75	2.884,54	bl		
HNG151	Visir	2.907,49	cv	1970	6
HNG152	Galant Carlberg	2.919,31	cv	1985	2
HNG153	Alf	2.951,70	cv	1978	2
HNG154	Breeding line 76	3.001,82	bl	**2012**	
HNG155	Danpro	3.012,44	cv	1969	2
HNG156	Otira	3.017,75	cv	1997	2
HNG157	Breeding line 77	3.031,57	bl		
HNG158	Ljubljana KVL 395	3.044,41	lr		2
HNG159	Grenoble I KVL 131	3.095,01	lr		6
HNG160	Breeding line 78	3.126,90	bl		
HNG161	Breeding line 79	3.143,85	bl		
HNG162	Fabel Sejet	3.219,70	cv	2001	2
HNG163	Bor 4	3.222,51	cv	2011	2
HNG164	Breeding line 80	3.292,72	bl		
HNG165	Breeding line 81	3.297,06	bl		
HNG166	Edvin	3.362,83	cv	2008	2
HNG167	Sort Glatstakket	3.383,38	lr		6
HNG168	Breeding line 82	3.465,03	bl		
HNG169	Osiris J-1277	3.480,84	lr		6
HNG170	Hafnia	3.565,60	cv	1958	2
HNG171	Breeding line 83	3.729,27	bl		
HNG172	Anakin	3.731,57	cv	2006	2
HNG173	Breeding line 84	3.756,24	bl		
HNG174	Breeding line 85	3.821,79	bl		
HNG175	Tartu KVL 349	3.852,89	lr		2
HNG176	Breeding line 86	3.864,31	bl		
HNG177	Freja	3.924,28	cv	1941	2
HNG178	Rasmusson	3.930,55	cv	2008	6
HNG179	Møyjar	4.003,41	cv	1969	2
HNG180	Cluj KVL 100	4.058,79	lr		6
HNG181	Breeding line 87	4.118,94	bl		
HNG182	Sebastian	4.132,03	cv	2002	2
HNG183	Breeding line 88	4.142,60	bl		
HNG184	Breeding line 89	4.186,85	bl		
HNG185	Magdeburg KVL 358	4.193,91	lr		2
HNG186	Tammi	4.255,87	cv	1937	4
HNG187	Breeding line 90	4.273,26	bl		
HNG188	Königsberg KVL 18	4.273,92	lr		6
HNG189	Drost Pajbjerg	4.308,74	cv	1951	6
HNG190	Breeding line 91	4.390,88	bl		2
HNG191	Keops	4.391,31	cv	2004	2

HNG192	Königsberg KVL 18	4.434,47	lr		6
HNG193	Laurikka	4.449,42	cv	2010	2
HNG194	Linus	4.535,95	cv	1997	2
HNG195	Fero	4.567,73	cv	1943	6
HNG196	NOS 16008-51	4.628,12	cv	2011	2
HNG197	Paavo	4.648,04	cv	1959	2
HNG198	Breeding line 92	4.649,77	bl	2013	
HNG199	Breeding line 93	4.675,26	bl		
HNG200	Breeding line 94	4.816,02	bl	2012	
HNG201	Orthega	4.822,19	cv	1997	2
HNG202	Vilm KVL 248	4.900,04	lr		2
HNG203	Breeding line 95	4.953,95	bl	2011	
HNG204	Breeding line 96	4.981,60	bl		2
HNG205	Breeding line 97	5.121,29	bl	2011	
HNG206	Alliot	5.197,35	cv	1999	2
HNG207	Probstei/Tabor KVL 362	5.233,95	lr		2
HNG208	Evergreen	5.313,33	cv	2010	6
HNG209	Szeged KVL 347	5.336,44	lr		2
HNG210	Harbinger	5.529,21	cv	2009	2
HNG211	Oslo KVL 25	5.562,74	lr		6
HNG212	Jacinta	5.739,13	cv	1998	2
HNG213	Birka	5.922,76	cv	1981	2
HNG214	NOS 15251-52	5.925,64	cv	2011	2
HNG215	Arla	6.172,27	cv	1962	2
HNG216	Ida	6.500,86	cv	1979	2
HNG217	Breeding line 98	6.740,28	bl		
HNG218	Laukko	6.908,78	lr		2
HNG219	Tartu KVL 349	7.059,76	lr		2
HNG220	Breeding line 99	7.281,33	bl		
HNG221	Metz KVL 124	7.366,26	lr		6
HNG222	Breeding line 100	8.087,67	bl		
HNG223	Breeding line 102	8.867,67	bl		
HNG224	Pavia KVL 386	8.941,29	lr		2
HNG225	Montpellier KVL 209	9.944,34	lr		2
HNG226	Breeding line 103	10.193,43	bl		
HNG227	Sanette	10.756,40	cv	2011	2

Supplementary Table S6. MRM transitions (positive ionization mode) and response factors for barley hydroxynitrile glucosides.

Analyte	Retention time [min]	Q1 [m/z]	Q3 [m/z]	Collision energy [eV]	Response factor[R]
Sutherlandin [M+H]+	1.20	276.2	114.1 Q	-14	0.15
			97.1	-24	
			69.1	-50	
Epidermin [M+H]+	1.58	262.1	204.0 Q	-10	1.27
			85.2	-19	
			163.0	-5	
			126.1	-17	
Osmaronin [M+H]+	1.69	260.1	98.1 Q	-14	0.07
			242	-10	
			70.2	-30	
Dihydroosmaronin [M+H]+	1.71	262.1	100.1 Q	-14	0.06
			142.1	-14	
			57.3	-24	
Epiheterodendrin [M+H]+	2.05	262.1	163.1 Q	-4	2.00
			85.2	-18	
			100.1	-14	

Q, quantifier ion; additional transitions are used for identification only. R; response factor compared to the internal standard amygdalin
